# Strategies for selection of subjects for sequencing after detection of a linkage peak

**DOI:** 10.1186/1753-6561-5-S9-S77

**Published:** 2011-11-29

**Authors:** Kristina Allen-Brady, James Farnham, Lisa Cannon-Albright

**Affiliations:** 1 Division of Genetic Epidemiology, Department of Internal Medicine, University of Utah, 391 Chipeta Way, Suite D, Salt Lake City, UT 84105, USA; 2 George E. Wallen Department of Veterans Affairs Medical Center, 500 Foothill Drive, Salt Lake City, UT 84148, USA

## Abstract

Linkage analysis has the potential to localize disease genes of interest, but the choice of which subjects to select for follow-up sequencing after identifying a linkage peak might influence the ability to find a disease gene. We compare nine different strategies for selection of subjects for follow-up sequencing using sequence data from the Genetic Analysis Workshop 17. We found that our more selective strategies, which included methods to identify case subjects more likely to be affected by genetic causes, out-performed sequencing all case and control subjects in linked pedigrees and required sequencing fewer individuals. We found that using genotype data from population control subjects had a higher benefit-cost ratio than sequencing control subjects selected as being the opposite extreme of the case subjects. We conclude that choosing case subjects for sequencing based on more selective strategies can be reliable and cost-effective.

## Background

The well-established genome-wide linkage analysis method has the potential to focus the search for and identification of genetic loci responsible for a disease or trait. Although linkage analysis has been successfully used to find high-penetrant rare loci for a number of different diseases, with the advent of high-density marker sets, the challenges associated with linkage analysis have limited its use somewhat. These challenges include (1) the difficulty and cost of ascertaining large high-risk pedigrees, (2) shifts in general thought about whether common complex diseases are caused by common variants (the common disease/common variant hypothesis, which has favored association study designs) or, most recently, by multiple rare variants [1], and (3) often the inability to localize and identify a disease gene under a previously established linkage peak.

In this study we focus on the third challenge, namely, the inability to identify a disease gene under a linkage peak. It is possible that the choice of subjects selected for follow-up sequencing after finding linkage evidence influences detection of underlying genetic loci. Because sequencing costs are still prohibitively expensive, particularly for large chromosomal regions, the number of subjects selected for follow-up sequencing may be limited. Here, we explore different strategies for selecting subjects for follow-up sequencing to identify the most reliable and cost-effective means for choosing appropriate sequencing candidates.

## Methods

### Data description

We use the mini-exome sequence data available for Genetic Analysis Workshop 17 (GAW17). Eight extended pedigrees are simulated based on gene dropping from the pedigree founders’ single-nucleotide polymorphism (SNP) genotypes, which were obtained from sequence alignment files provided by the 1000 Genomes Project pilot3 study. The original files include exonic genotype data for 3,205 genes and 24,487 SNPs. Eight multilineal pedigrees are provided and include 697 individuals, all of whom are genotyped. For each of the 697 individuals, we are also provided with information on affection status, age, sex, smoking status, and three quantitative trait variables called Q1, Q2, and Q4. According to the answers provided by the GAW17 data simulators, disease risk is influenced directly by age, smoking status, Q1, Q2, and Q4 and indirectly by sex through Q4. There are 200 replicates of the phenotype data variables; genotype data are constant over the 200 replicates.

### Subjects and linkage subset of markers

We split the pedigrees into 23 unilineal pedigrees with the intent to include as many individuals in a pedigree as possible. Because multiple genes were known to contribute to the disease phenotype, we split pedigrees to reduce intrafamilial genetic heterogeneity. No duplication of individuals was allowed; parents of some individuals were set to zero to break links. After splitting, our data set for each replicate contained 648 individuals. We determined a linkage subset of markers on the basis of marker heterozygosity (minimum heterozygosity = 0.1) and linkage disequilibrium (minimum *r*^2^ = 0.16). Heterozygosity was calculated as 1 − (*p*^2^ + *q*^2^), where *p* is the major allele frequency and *q* is the minor allele frequency. We calculated linkage disequilibrium between all pairs of markers in pedigree founders using PLINK [2] and a 100-Mb window.

### Weighting of case subjects and linkage model

Our outcome phenotype was affection status. Based on the GAW17 answers, we performed a logistic regression analysis and included as predictors of affection status Age, Sex, Smoking status, and the three quantitative variables. Using the generated predictive probabilities from the logistic regression model, we correctly predicted affection status for approximately 90% of the case subjects (depending on the replicate) using a cut point of 0.5. By graphing the predicted probabilities, we divided case subjects and control subjects into four quadrants based on case and control status and predictive probability (<0.5 or ≥0.5). Liability classes for the linkage analysis were divided into these four categories. Control subjects with low predictive probability (low environmental scores) and case subjects with high predictive probability (high environmental scores) were assigned a weaker penetrance function. Control subjects with high predictive probability (high environmental scores), which we call high-covariate control subjects, and case subjects with low predictive probability (low environmental scores), which we call low-covariate case subjects, were assigned a more powerful penetrance function.

We performed a parametric model-based analysis using general dominant and recessive models. For the dominant model, we used a minor allele frequency of 0.01; for carriers of 0, 1, or 2 copies of the rare allele, the weaker penetrance function was set to be 0.2, 0.6, 0.6, respectively, and the more powerful penetrance function was set to be 0.0005, 0.5, 0.5, respectively. For the recessive model, we used a minor allele frequency of 0.01; for carriers of 0, 1, or 2 copies of the rare allele, the weaker penetrance function was set to be 0.2, 0.2, 0.6, respectively, and the more powerful penetrance function was set to be 0.0005, 0.0005, 0.5, respectively.

### Linkage analysis

Linkage analysis was performed using the multipoint Markov chain Monte Carlo linkage method MCLINK [3], which allows for multipoint linkage analysis of large extended pedigrees. Linkage results for MCLINK have been shown previously to produce similar results to other linkage packages [4]. Because the phenotype is known to be attributable to multiple different genes, we report heterogeneity LOD (HLOD) scores. MCLINK also provides information on the most likely haplotype configuration from the observed data; we assumed that the haplotype of interest was the haplotype carried by the greatest number of case subjects in a linked pedigree. We estimated allele frequencies from observation of genotype data from all individuals. For the genetic map, we assumed that 1 Mb was equivalent to 1 cM. This simple assumption has been shown to produce linkage results that are generally identical with a more detailed genetic map [5]. To generate sufficient power to detect linkage evidence, we summed the HLOD scores for the 23 pedigrees across 10 replicates of data. Each linkage analysis data replicate was completed as described earlier. We used the Lander and Kruglyak criteria to determine significant (HLOD > 3.3) and suggestive (HLOD > 1.86) evidence for linkage [6]. Pedigrees linked to a region of interest were defined as having nominally significant linkage evidence if their LOD score was greater than 0.588, which corresponds to a *p*-value of 0.05, not accounting for multiple testing.

### Follow-up association testing

After identification of linkage evidence and pedigrees linked to a region, we performed association testing for only the subjects in the linked pedigrees, using Genie [7], a software tool that accounts for the relatedness of subjects using Monte Carlo simulation. A single disease-causing variant, identified using the GAW17 answers, was tested in the association analyses. We compared two types of control samples to the case samples. For the first set of control samples, we selected all founders in the original eight pedigrees to serve as population controls. Because the disease was common in the population, a high percentage of these individuals were case subjects, but use of their genotype frequencies allowed for a consistent comparison group across the various case groups studied, despite the conservative results that were expected. The second type of control sample was selected to be, in most cases, the opposite extreme of the case group. For the second group of control samples, all case subjects and control subjects were selected from the linked pedigrees. Because the number of case and control subjects is often small, this selection resulted in some cell counts being zero. To these zero cells we added 0.5 in order to calculate an odds ratio. If the samples were all from different pedigrees, then significance was determined using Fisher’s exact test in PLINK [2]. However, if the selected case subjects or control subjects were related and there was a cell with a zero frequency, we were not able to calculate the significance because Genie [7] does not perform Fisher’s exact test.

### Association strategies

We compared nine different strategies for selection of case subjects for sequencing in the eight linked pedigrees (see detailed list in Tables 1 and 2). Two of the strategies focused on age because earlier onset is more likely to be genetic. Four of the strategies focused on the low-covariate status because subjects in these groups were at decreased risk for environmental contributions to their disease and at greater risk for genetic contributions. In these two subgroups we also examined individuals who were haplotype carriers. Finally, we looked at three additional strategies: examining all haplotype carriers, examining all case subjects, and selecting a random case from each pedigree. Our intent was to compare selective strategies, for which case subjects had a stronger genetic contribution to their disease, with more general strategies, such as selecting all case subjects or a random case subject from each pedigree.

### Comparison of association strategies

Because sample size differed between each of the nine case selection scenarios explored, we compared odds ratio effect sizes between the strategies so that the power of each test would not influence our results. To compute a benefit-cost ratio for each strategy, we assumed that the benefit of each strategy was the odds ratio effect size and that the cost was the total number of subjects genotyped. For the population control set (Table 1), we included the cost of the case subjects only; we assumed that the founder control subjects represented a population sample and that those data would be available publicly. For the opposite extreme matched control subjects (Table 2), we assumed that both the case group and the opposite extreme control group would need to be genotyped, and hence both groups were included in the cost calculation.

## Results

We identified two regions that met the criteria for suggestive evidence of linkage, and using the GAW17 answers, we identified one of them as harboring a disease gene. The linkage peak identified was on chromosome 17 between C17S3663 and C17S5325 under a dominant model; our peak HLOD score was 2.44 at marker C17S5244. From the answers, we identified a disease gene, *PRKCA*, that affected disease liability with casual variants C17S4578 and C17S4581. Eight pedigrees out of 230 pedigrees tested (23 split pedigrees across 10 replicates) had individual LOD scores greater than 0.588. Variation existed for C17S4578, but not C17S4581, across the eight pedigrees. Association testing proceeded for C17S4578 using individuals from the eight linked pedigrees.

In Table 1 we show the results using the 202 founders as the reference population, and in Table 2 we give the results for control subjects selected to be the opposite extreme of the case samples. Overall, the more selective strategies (i.e., strategies A–G in the tables) resulted in greater effect sizes and higher benefit-cost ratios than the more general strategies (strategies H and I in the tables). The strategy of selecting the youngest haplotype-carrier case per pedigree (strategy A) had the highest overall effect size and the highest benefit-cost ratio in Table 1. In Table 2, selection of all low-covariate haplotype-carrier case subjects and all high-covariate non-haplotype-carrier control subjects (strategy C) performed best overall in terms of effect size and benefit-cost ratio. In general, the top three strategies (i.e., haplotype carriers selected as being the youngest [A] or as having low covariates [C] or selection of the youngest case per pedigree [B]) performed well across one of the two types of control populations or inheritance models. We note in particular that the all low-covariate haplotype-carrier case subjects (strategy C) performed consistently well across both dominant and recessive models and both types of control populations.

**Table 1:** Association analysis for C17S4578 comparing case subjects from 8 linked pedigrees to 202 original pedigree founders

Strategy	Case subjects (*n*)	Case genotype counts^a^	Control subject genotype counts^a^	Dominant model		Recessive model	
	
				Effect size	Benefit-cost ratio	Effect size	Benefit-cost ratio
Strategies focused on age							
A. Youngest haplotype-carrier case per pedigree	8	5/1/2	15/100/87	2.27	0.28	20.78^c^	2.60
B. Youngest case per pedigree	8	4/4/0	15/100/87	12.1^b,c^	**1.51**	12.47^c^	1.56
Strategies focused on low-covariate status							
C. All low-covariate haplotype-carrier case subjects	13	7/5/1	15/100/87	9.08^c^	0.70	14.54^c^	1.12
D. Families with 2+ low-covariate haplotype-carrier case subjects	9	4/4/1	15/100/87	6.05	0.67	9.97^c^	1.11
E. All low-covariate case subjects	16	7/6/3	15/100/87	3.28	0.21	9.7^c^	0.61
F. Lowest covariate case subject per pedigree	8	3/4/1	15/100/87	5.3	0.66	7.48^c^	0.94
Other strategies							
G. All haplotype-carrier case subjects per pedigree	40	10/21/9	15/100/87	2.61	0.07	4.16^c^	0.10
H. All case subjects	81	10/40/31	15/100/87	1.22	0.02	1.76	0.02
I. Random case subject per pedigree	8	0/5/3	15/100/87	1.26	0.16	0	0.00

^a^ The three values are the counts for homozygote rare, heterozygote, and wild-type variants.

^b^ For cells with zero count, we added 0.5 to the cell to compute the odds ratio.

^c^*p* < 0.05.

**Table 2:** Association analysis for C17S4578 comparing case subjects to opposite extreme control subjects in eight linked pedigrees

Strategy	Case subject genotype counts^a^	Control subject genotype counts^a^	Dominant model		Recessive model	
	
			Effect size	Benefit-cost ratio	Effect size	Benefit-cost ratio
Strategies focused on age						
A. Youngest haplotype-carrier case subject in each pedigree vs. oldest non-haplotype-carrier control subject	5/1/2	1/5/2	1	0.06	11.67	0.73
B. Youngest case subject vs. oldest control subject in each pedigree	4/4/0	1/3/4	16.0^b^	**1.0**	7	0.44
Strategies focused on low-covariate status						
C. All low-covariate haplotype-carrier case subjects vs. all high-covariate non-haplotype-carrier control subjects	7/5/1	0/4/6	18^c^	0.78	23.3^b,d^	1.01
D. Families with 2+ low-covariate haplotype-carrier case subjects vs. all high-covariate non-haplotype-carrier control subjects	4/4/1	0/4/6	12^c^	0.63	16.0^b,d^	0.84
E. All low-covariate case subjects vs. all high-covariate control subjects	7/6/3	0/4/7	7.58	0.28	17.1^b,d^	0.63
F. Lowest covariate case subject vs. highest covariate control subject per pedigree	3/4/1	0/4/4	7	0.44	5.3	0.33
Other strategies						
G. All haplotype-carrier case subjects vs. all non-haplotype-carrier control subjects per pedigree	10/21/9	7/66/104	4.91^c^	0.02	8.1^c^	0.04
H. All case subjects vs. all control subjects	10/40/31	10/86/110	1.85^c^	0.01	2.76^c^	0.01
I. Random case subject vs. random control subject per pedigree	0/5/3	1/2/5	2.78	0.17	0	0.00

^a^ The three values are the counts for homozygote rare, heterozygote, and wild-type variants.

^b^ For cells with zero count, we added 0.5 to the cell to compute the odds ratio.

^c^*p* < 0.05.

^d^ Unable to compute significance because of a zero cell, and either case or control subjects or both are related.

In general, we observed that the overall benefit-cost ratio was higher under both a dominant and a recessive model using the 202 founders as the control population, because of the increased power obtained using a larger control group and the additional benefit of not incurring a cost to genotype the control specimens. However, the effect size for both the dominant and recessive models tended to be higher when the opposite extreme control subjects were used as the comparison group (i.e., results in Table 2). This is expected because the disease was common among the 202 population controls.

## Discussion and conclusions

Linkage analysis has the benefit of being able to localize disease genes of interest and to identify which pedigrees should be studied for follow-up sequencing to find the disease genes. Here, we examined nine strategies for selecting subjects for follow-up sequencing. Because sequencing costs are still relatively expensive, it is imperative to choose subjects for sequencing who will most likely carry variants in the disease gene.

We found that all the more selective strategies, such as selecting subjects based on covariate and haplotype information, out-performed general strategies of sequencing all case subjects in linked pedigrees or sequencing a random case subject in linked pedigrees. In the linked pedigrees many case subjects are sporadic cases. Effect sizes are increased when more case subjects likely to have a genetic contribution to their disease are included in an analysis, but effect sizes are not increased when more sporadic case subjects are included.

We found that the most consistently reliable strategy across both dominant and recessive models and across the two types of control groups was selection of haplotype carriers who were classified as low-covariate (strategy C) from regression analysis. For this GAW17 analysis, the data simulators provided us with the covariate risk factors for the disease. It was our intent to find subjects who had the disease but whose disease was more likely attributable to genetic causes. This strategy can be applied to many complex diseases. Although all risk factors may not be known for a particular disease, major risk factors are likely to be known and could be incorporated into both the linkage model and the subsequent selection strategy for identification of sequencing candidates. We also saw good success with selection of the youngest haplotype-carrier case subject or the youngest case subject per pedigree. Use of early-onset case subjects as a surrogate for the low-covariate strategy may be useful when major risk factors for a disease are not known.

In this study, we also explored two types of control groups: founders from the original pedigrees, which represented population controls, and control subjects selected as being the opposite extreme of the case subjects. We found that the benefit-cost ratio was higher, in general, across both dominant and recessive models for the population control group than for the model in which control subjects were selected to be the opposite extreme of case subjects. The assumption that population control data can be obtained in the future without cost is reasonable, because genotype data can be obtained freely now for various cohorts (e.g., Illumina iControl data and soon the 1000 Genomes Project). However, it should be noted that if a disease is common in the population, results using publicly available sequence data will be conservative.

We identified two linkage peaks that had suggestive evidence of linkage, only one of which contained a variant of interest. Suggestive evidence is defined as a LOD score expected once per chance per genome scan [6]. Hence finding a suggestive linkage result that turns out to be a false-positive result is within expectation.

In conclusion, choosing case subjects for sequencing based on being a haplotype carrier and having low-covariate status can be reliable and cost-effective.

## Competing interests

The authors declare that there are no competing interests.

## Authors’ contributions

KAB: Designed the study, performed the statistical analyses and drafted the manuscript. JF: Assisted with data analysis, attended the GAW17 meeting and assisted with manuscript preparation. LCA: Conceived of the study and helped to draft the manuscript. All authors read and approved the final manuscript.
